# Assessing the impact of common pain medications on gut microbiota composition and metabolites: insights from a Mendelian randomization study

**DOI:** 10.1099/jmm.0.002028

**Published:** 2025-06-12

**Authors:** Feng Wei, Diefei Liang, Junxiong Qiu, Yuan Fu, Zhaopei Zeng, Jiarui Zhang, Xinyi Zhang, Jianwei Lin, Junmeng Zheng, Liling Lin

**Affiliations:** 1 Department of Cardiovascular Surgery, Sun Yat-sen Memorial Hospital, Sun Yat-sen University, Guangzhou, PR China; 2 Department of Endocrinology, Sun Yat-sen Memorial Hospital, Sun Yat-sen University, Guangzhou, PR China; 3 Big Data Laboratory, Joint Shantou International Eye Center of Shantou University and The Chinese University of Hong Kong, Shantou, Guangdong, PR China; 4 Department of Anesthesiology, Sun Yat-sen Memorial Hospital, Sun Yat-sen University, Guangzhou, PR China

**Keywords:** analgesics, causal association, circulating metabolites, gut microbiota composition, Mendelian randomization

## Abstract

**Introduction.** The relationship between analgesic use and gut microbiota alterations has garnered increasing attention. However, the causal link between these two factors remains to be elucidated. Given the prevalence of analgesic use and the significant role of gut microbiota in human health, clarifying this relationship is of great importance.

**Hypothesis/Gap Statement.** Existing observational studies are limited in their ability to establish causality between analgesic use and gut microbiota alterations. Therefore, there is a need for robust causal inference methods to explore this relationship and uncover the underlying mechanisms.

**Aim.** This study aims to investigate the causal associations between genetic susceptibility to four common analgesics (NSAIDs, salicylic acid, opioids, and anilides) and gut microbiota composition, as well as circulating metabolites, using a two-sample Mendelian randomization approach.

**Methodology.** A two-sample Mendelian randomization was used to investigate the potential association between genetic susceptibility to four analgesic uses and gut microbiota composition, as well as circulating metabolites. Summary-level statistics of genome-wide association studies were obtained from primarily European ancestry cohorts, including 466,457 participants from the UK Biobank and 18,340 individuals from the MiBioGen consortium.

**Results.** Only one suggestive causal association was found between NSAID use and elevated abundance of gut microbiota, namely group *Eubacterium xylanophilum*. In addition, salicylic use was correlated with an increased abundance of the family *Prevotellaceae* (*P*=0.006)*,* while it was negatively associated with the abundance of 8 microbiota traits, including genus *Clostridiumsensustricto1*, *Adlercreutzia*, *Akkermansia*, family *Clostridiaceae1*, *Verrucomicrobiaceae*, phylum *Verrucomicrobia*, class *Verrucomicrobiae* and order *Verrucomicrobiales* with *P* value ranging from 0.009 to 0.043. No clear evidence was found between opioid and anilide use and gut microbiota alteration. Meanwhile, salicylic use was potentially causally associated with four metabolites, including acetoacetate, creatinine, omega-3 fatty acids and triglycerides in very large high-density lipoprotein, with *P* values ranging from 0.005 to 0.046.

**Conclusion.** The results of this study offer powerful evidence that the long-term use of salicylic acid may substantially impact gut microbiota composition and circulating metabolites. Further investigations are needed to uncover the underlying mechanisms.

## Data Availability

The datasets and related database links used in this study were fully announced and listed in number tables. The datasets used to support the conclusions of this paper were presented in the article and its accompanying files.

## Introduction

In recent years, with the growing focus on the study of gut microbes and their metabolic mechanisms, more and more scholars believe that the gut microbiota has a significant part to play in the development and progression of human diseases [1,2]. Some findings suggest that intestinal dysfunction increases the risk of developing various disorders [3]. During the investigation into the regulatory pathways and mechanisms of neurological disorders induced by gut microbes, the concept of the brain–gut axis has emerged and is garnering significant attention [4]. It is well known that the brain–gut axis is a cross-talk between the enteric nervous system and the central nervous system (CNS) [5,6]. Among the myriad regulatory functions attributed to the brain–gut axis, the modulation of gut microbiota has become a focal point of interest [7,8].

Chronic pain is now recognized as a distinct disease [9]. Inadequate treatment of chronic pain can have far-reaching consequences, contributing to the development of multiple systemic disorders, including cardiovascular disease [10]. The utilization of a range of commonly prescribed analgesics continues to be the primary approach for chronic pain relief, including opioids, anilides, NSAIDs and salicylic acid [11,12]. Several previous studies have shown that different clinical analgesics have diverse collateral effects on the gastrointestinal (GI) tract when exerting analgesic effects [13,14]. Typically, opioids and NSAIDs tend to cause psychiatric dependence and gastrointestinal inflammation [15,17]. Similarly, salicylic acid possesses not only beneficial anti-inflammatory, antipyretic and analgesic properties but also exerts a potent stimulatory effect on the GI mucosa [18,19]. In addition, the presence of analgesic receptors in the GI tract and CNS suggests that common analgesics may exert relevant effects by modulating the brain–gut axis [20,21].

As an increasing number of observations are being gathered, a potential link has been recognized between the long-term use of painkillers, alterations in gut microbiota composition and changes in metabolites [22,23]. However, the presence of confounding factors and the possibility of reverse causality make it unclear whether there is a causality between the above three factors. Given the crucial role of the brain–gut axis and the indispensability of analgesic medications in managing chronic pain, it is of paramount importance to assess the specific impact of a particular painkiller on the gut microbiota. This evaluation holds significant value in guiding clinical decision-making.

The Mendelian randomization approach uses single nucleotide polymorphisms (SNPs) as the instrumental variables to infer causal relationships between exposures and outcomes, which can effectively overcome the possible bias caused by confounding factors and reverse causality [24,25]. In this research, the Mendelian randomization approach was used to infer the associations between analgesic use, alteration in the composition of gut microbiota and metabolites.

## Methods

### Study design overview

**Fig. 1.**
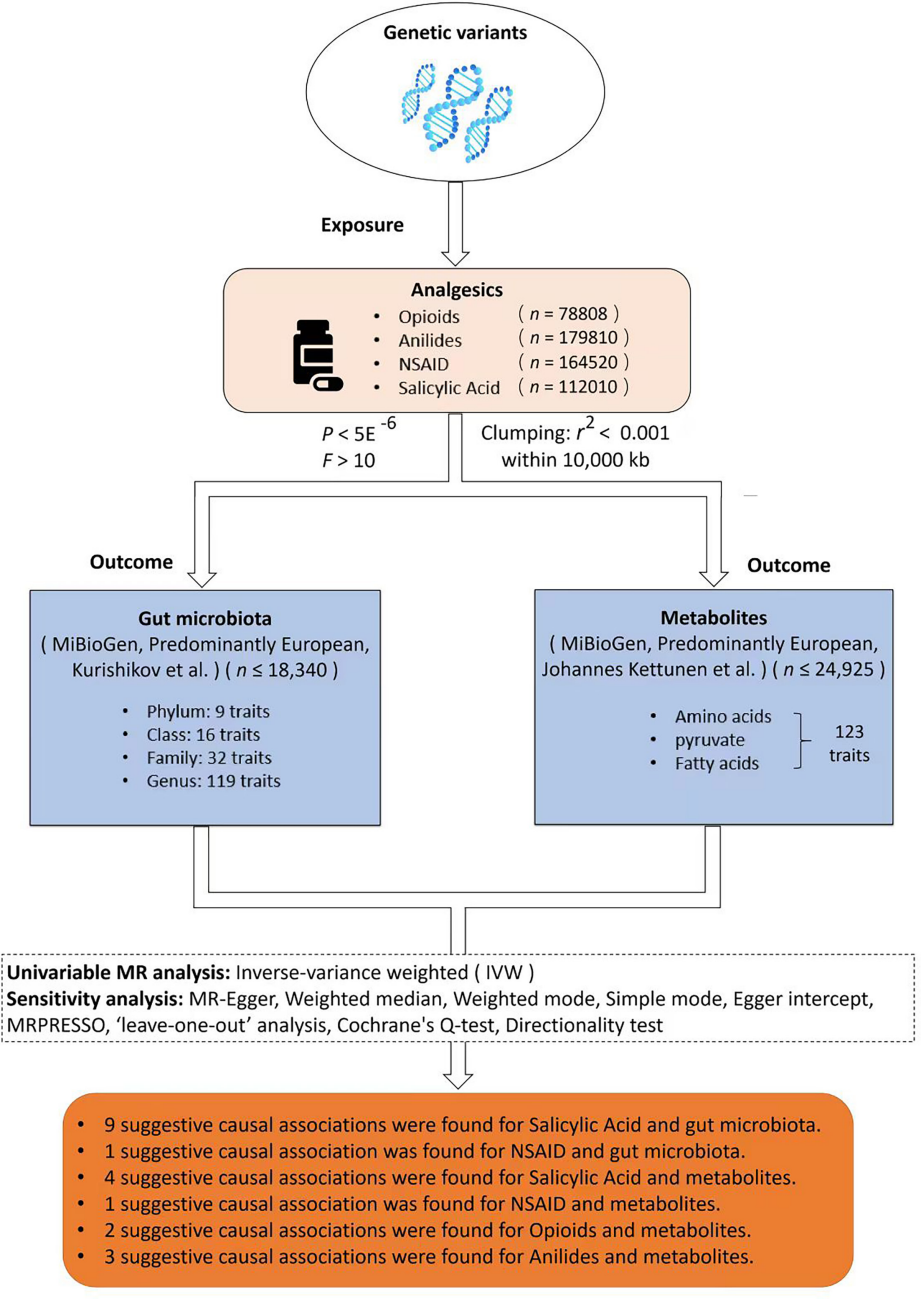
Flow chart of study design. This is a two-sample MR study to analyse and evaluate potential causal associations between four painkillers and gut microbiota composition, as well as gut circulating metabolites. SNP data related to the four analgesics, including anilides, opioids, NSAID and salicylic acid, are conditionally screened and used as instrumental variables for common exposure factors in this two-sample MR. Then, MR analysis and evaluation of the potential causal relationship between painkillers and gut microbiota, as well as metabolites, were carried out using IVW as the primary method and various methods, including sensitivity analysis. Finally, the potential causal association between the genetic variation of the four painkillers and gut microbiota, as well as metabolites, was shown in the above flow chart after MR.

A two-sample Mendelian randomization (MR) framework was applied in the research to evaluate the causal relationships between four common analgesics and the composition of gut microbiota. A sensitivity analysis was followed to provide robust MR results. An additional MR analysis was performed to rule out potential impacts of confounders of both exposure and outcome (analgesic drugs and gut microbiota) by excluding SNPs that are associated with these confounders. A reverse MR was carried out on the positive findings to see whether gut microbiota also has an impact on the liability of analgesic drug use. This study was conducted and reported in accordance with the STROBE-MR statement [26,27]. The flow chart of the study design is shown in Fig. 1.

The basic principles of MR are based on three assumptions. Hypothesis 1: Genetic variants are strongly associated with the exposure of interest. Hypothesis 2: Genetic variants are independent of the confounding of the exposure and outcome. Hypothesis 3: Genetic variants are not relevant to the outcomes of interest.

### Data sources for pain medications

Summary data for pain medication use were obtained from genome-wide association studies (GWASs) based on 318,177 individuals of European ancestry from the UK Biobank [28]. Wu *et al.* conducted GWASs of self-reported regular medication use from 23 categories [28], among which, 4 are pain medications, including opioids (eg, morphine, oxycodone, codeine, fentanyl, pethidine and tramadol), NSAIDs and anilides, as well as salicylic acid and derivatives [29]. Analgesic drugs are categorized through a method performed by the effective ingredient in the Anatomical Therapeutic Chemical Classification System [30]. The medication GWAS analyses were conducted with adjustment of age, sex, assessment centre and 20 genetic principal components. Since limited loci reached the genome-wide significance, we relaxed the selection criteria at the *P* threshold of 5×10^−6^ to include more SNPs. Detailed information for pain medications is shown in Table S1, available in the online Supplementary Material.

### Data sources for gut microbiota

The summary data for gut microbiota was leveraged from MiBioGen, the largest GWAS for gut microbiota thus far [31]. The MiBioGen consortium curated and analysed genome-wide genotypes and microbiome data from 18,340 individuals (24 cohorts, predominantly European ancestry) using faecal 16S rRNA gene sequencing. The summary data were adjusted for covariates. A total of 211 bacteria were yielded, including 9 phyla, 16 classes, 20 orders, 35 families and 131 genera. Unclassifiable microbiota traits were excluded, and we were left with 9 phyla, 16 classes, 20 orders, 32 families and 119 genera for further analysis. Due to the limited genetic loci studied, we relaxed the inclusion criteria to *P* < 1×10^−5^ as recommended by Sanna *et al.* [32]. The summary statistics for metabolites were leveraged from Johannes *et al.*, where 123 circulating metabolic traits were studied.

### Statistical analysis

As described in the data sources for the original GWAS, samples with overlap between exposures and outcomes were negligible in this research. To ensure the independence of instrumental variables, we clumped the SNPs with more stringent linkage disequilibrium (LD) criteria at *r*^2^ >0.001, within a 10,000 kbp window using the European ancestry reference panel of the 1,000 Genomes Project [33]. SNP effects and relevant standard errors were gained from GWASs of exposures and outcomes, respectively. To avoid weak instrumental variable bias, we calculated the F-statistics of each SNP and removed those SNPs with F-statistics less than 10. We then harmonized the exposure and outcome data, removing the palindromic SNPs with intermediate allele frequencies. The Steiger filtering was also applied to the harmonized data to identify and filter out SNPs showing reverse causality from the assay reports. A reverse causation is defined as the observed variance of the outcome exceeding the observed variance of the exposure [34]. SNPs that were not available in the outcome dataset were removed.

After instrumental variable selection, MR analyses were carried out using the inverse-variance weighted (IVW) method as the primary method. The IVW method offers enhanced efficiency by generating more precise estimates across various scenarios [35]. To provide robust MR evidence, four additional methods, including MR-Egger, weighted median, simple mode and weighted mode, were applied as sensitivity analyses [34]. The MR-Egger intercept and MRPRESSO were adopted for the horizontal pleiotropy test [35,36]. The SNP heterogeneity was tested by Cochran’s *Q* test and leave-one-out analysis [37]. Finally, the MR Steiger was applied to see whether the causal direction was correct [38].

The beta value and its 95% confidence intervals (CIs) were employed to represent the MR estimates of per log odds of the analgesic medication use for the change in abundance of gut microbiota. Benjamini–Hochberg’s false discovery rate (FDR) was used for multiple comparisons, and an FDR-corrected *P* value less than 0.05 was deemed significant. Association with nominal *P*<0.05 but greater than the FDR-corrected threshold was considered a suggestive association.

All MR analysis was carried out using the TwoSampleMR package (version 0.5.6) and the MRPRESSO package (version 1.0) in the software version R4.1.2 computing environment.

## Results

A total of 18,179 SNPs were identified for medication use at the selection threshold of *P*<5×10^−6^, including 5,908 for opioids, 4,633 SNPs for anilides, 4,187 SNPs for NSAID and 3,451 SNPs for salicylic acid use. After clumping and removing SNPs at LD *r*^2^ >0.001 within a 10,000 kbp window and SNPs that represent reverse causality as well as weak instrumental variables, 35 independent SNPs for opioids, 37 SNPs for anilides, 38 SNPs for NSAID and 40 SNPs for salicylic acid use were left as instrumental variables, respectively, as shown in Tables S1 and S2.

### Associations between genetic liability to anilide use and gut microbiota

No clear evidence was found for the causal association between anilide use and gut microbiota.

### Associations between genetic liability to opioid use and gut microbiota

No clear evidence was found for the causal association between opioids use and gut microbiota.

### Associations between genetic liability to NSAID use and gut microbiota

**Fig. 2.**
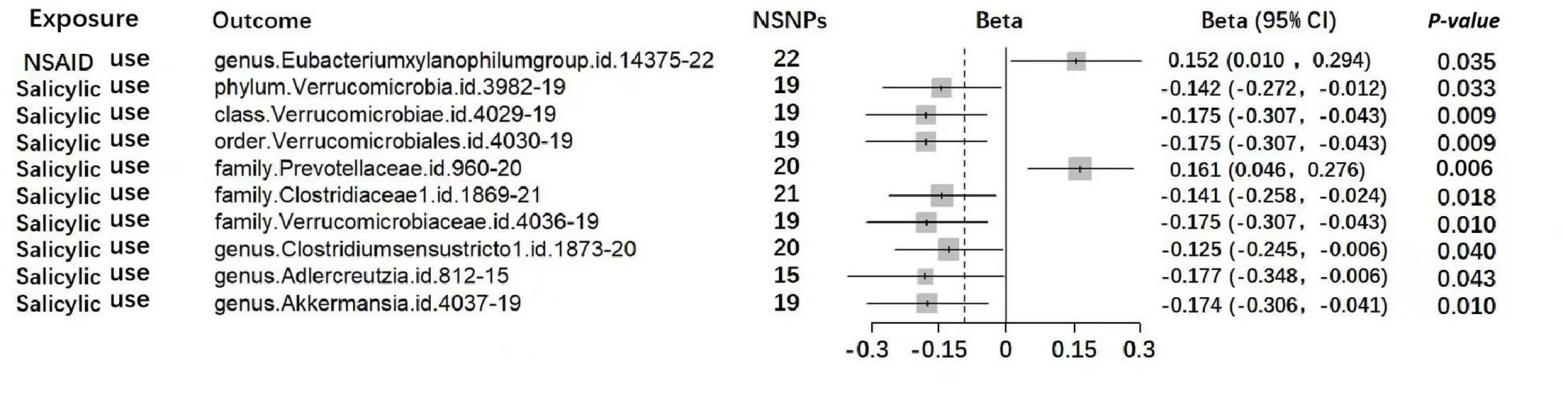
Forest plot of MR estimates between analgesics and gut microbiota. Causal effects from the inverse-variance weighted Mendelian randomization method. Effect estimates with *β* and its 95% CI were used based on the continuity of outcomes. Estimates for each 10-unit increase in gut microbiota following salicylic use. NSNPs, number of SNPs.

Only one suggestive causal association was found between NSAIDs and gut microbiota among all 196 traits. Per log odds ratio increase in NSAID use was associated with the elevated abundance of Eubacteriumxylanophilumgroup.id.14375 [beta=0.152, 95% CI = (0.01, 0.294) *P*=0.035, FDR-*P*=0.997] using the IVW method (Fig. S1). The MR estimates were consistent across four different methods, including weighted median, weighted mode, simple mode and MR-Egger. Apart from this, no other gut microbiota was observed to be significantly affected by NSAID from the phylum to the genus level in this study (Fig. 2, Table S4).

### Associations between genetic liability to salicylic acid use and gut microbiota

In this study, the impact of salicylic acid use on the abundance of gut microbiota is the most extensive among all the analgesics included in the study. At the genus level, we found that salicylic acid resulted in a reduced relative abundance of Clostridiumsensustricto1.id.1873 [beta=−0.125, 95% CI = (−0.245, –0.006), *P*=0.04], Adlercreutzia.id.812 [beta=−0.177, 95% CI = (−0.348, –0.006), *P*=0.043] and Akkermansia.id.4037 [beta=−0.174, 95% CI = (−0.306, –0.041), *P*=0.01] by the IVW method. Similarly, causal associations were found between salicylic use and reduced relative abundance of family Clostridiaceae1.id.1869 [beta=−0.141, 95% CI = (−0.258, –0.024), *P*=0.018] and family Verrucomicrobiaceae.id.4036 [beta=−0.175, 95% CI = (−0.307, –0.043), *P*=0.01]. Meanwhile, salicylic was found to be correlated with the increased abundance of family Prevotellaceae.id.960 [beta=0.161, 95% CI = (0.046, 0.276), *P*=0.006]. Notably, genetic liability to salicylic acid use was found to be associated with the reduced abundance of the *Verrucomicrobiae* family, including Verrucomicrobiae.id.4029 [beta=−0.175, 95% CI = (−0.307, –0.043), *P*=0.009], Verrucomicrobiales.id.4030 [beta=−0.175, 95% CI = (−0.307, –0.043), *P*=0.009] and Verrucomicrobia.id.3982 [beta=−0.142, 95% CI = (−0.272, –0.012), *P*=0.033] at the phylum, order and order levels, respectively (Fig. 2, Table S4).

### Associations between genetic liability to anilide use and circulating metabolites

**Fig. 3.**
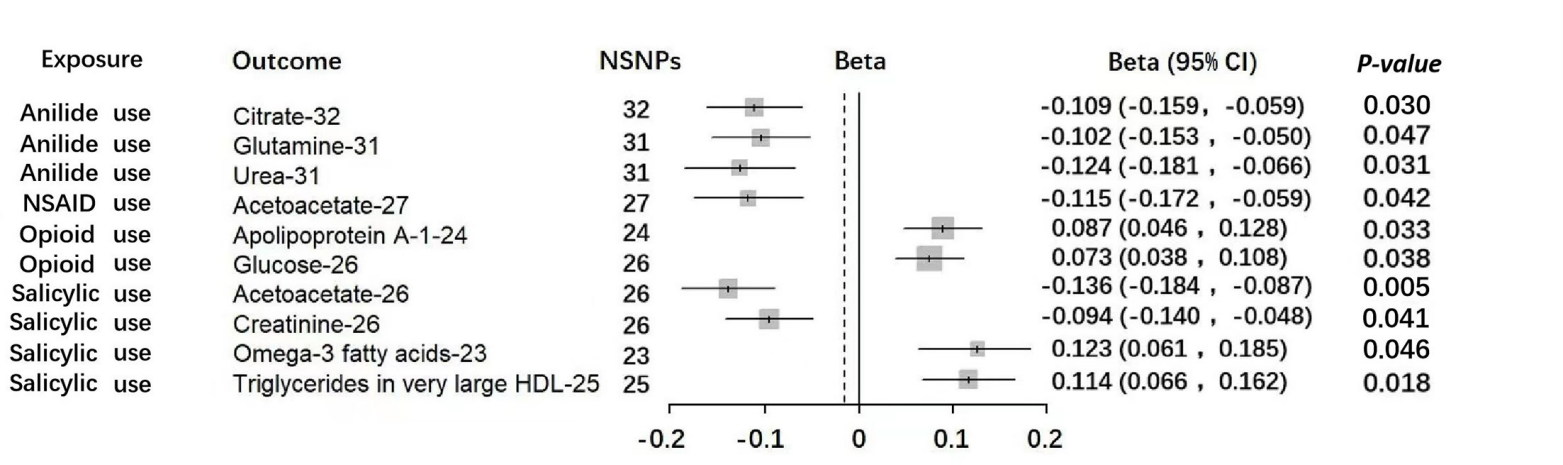
Forest plot of MR estimates between analgesics and circulating metabolites. Using a similar MR approach as described above, estimates for each 10-unit increase in gut circulating metabolites following four painkiller uses. NSNPs, number of single nucleotide polymorphisms.

Genetic liability to anilide use was found to have an effect not only on the abundance of gut microbiota but also on the levels of metabolites. Potential causal relationships were observed between anilide use and three metabolites, including citrate [beta=−0.109, 95% CI = (−0.159, –0.059), *P*=0.030], glutamine [beta=−0.102, 95% CI= (−0.153, –0.050), *P*=0.047] and urea [beta=−0.124, 95% CI = (−0.181, –0.066), *P*=0.031] (Fig. 3, Table S5).

### Associations between genetic liability to opioid use and circulating metabolites

The levels of two circulating metabolites were found to be affected by the genetic liability to opioid use, including apolipoprotein A-I [beta=0.087, 95% CI = (0.046, 0.128), *P*=0.033] and glucose [beta=0.073, 95% CI = (0.038, 0.108), *P*=0.038] (Fig. 3, Table S5).

### Associations between genetic liability to NSAID use and circulating metabolites

Only one potential causal relationship was found between NSAID use and the levels of Acetoacetate [beta=−0.115, 95% CI = (−0.172, –0.059), *P*=0.042] (Fig. 3, Table S5).

### Associations between genetic liability to salicylic acid use and circulating metabolites

Genetic liability to salicylic acid use exhibited a wide range of implications in terms of circulating metabolites, such as acetoacetate [beta=−0.136, 95% CI = (−0.184, –0.087), *P*=0.005], creatinine [beta=−0.094, 95% CI = (−0.14, –0.048), *P*=0.041], omega-3 fatty acids [beta=0.123, 95% CI = (0.061, 0.185), *P*=0.046] and triglycerides in very large high-density lipoprotein (HDL) [beta=0.114, 95% CI = (0.066, 0.162), *P*=0.018] (Fig. 3, Table S5).

### Sensitivity analysis

The test for pleiotropy using Egger intercept showed no significant horizontal pleiotropy (*P*>0.05), followed by the MR-PRESSO global test for pleiotropy of the four analgesics and gut microbiota (*P*＞0.05). Subsequently, Cochrane’s *Q* tests did not detect significant heterogeneity in painkillers, with *I*^2^ ranges below 17%, as shown in Tables S4 and S5. The scatterplots provided a sufficient basis for the unbiased and dependable results (Figs S1 and S2). Meanwhile, any SNP with extreme values was largely certified using the leave-one-out method (Figs S3 and S4). No significant difference was observed in determining the *P* values of the association between gut microbiota and analgesics and using MR estimates, suggesting that the included confounding factors did not substantially affect causality. Also, four analgesics were evaluated for their risk to gut microbiota and circulating metabolites (*P*<0.05). For the significance of causality, it was found that MR power was all greater than 80% at beta=0.3 (Table S3). However, insufficient detection power could not be fully ruled out when the MR estimates were relatively small.

## Discussion

In this study, an MR framework was applied to evaluate the causal link between genetic susceptibility to the utilization of four common painkillers and the composition of gut microbiota as well as metabolites. The findings did not yield conclusive evidence for a causal relationship between the use of anilides and opioids and gut microbiota. However, there was one potential causal association observed between NSAIDs use and gut microbiota, along with nine potential causal associations between the use of salicylic acid and gut microbiota. Moreover, each class of prescription painkiller exhibited distinct impacts in terms of metabolites. Specifically, two potential causal associations were found between opioid use and metabolites, three potential causal associations were identified between anilide use and metabolites, four potential causal associations were observed between salicylic use and metabolites and one potential causal association was found between salicylic use and metabolites.

Based on the results of an assessment of the causal relationship between analgesics and gut microbes using MR, we concluded that salicylic acid appeared to have a broad influence. First, we found that salicylic acid was associated at the genus level with the lower relative abundance of Adlercreutzia.id.812, Akkermansia.id.4037 and Clostridiumsensustricto1.id.1873. Also at the family level, salicylic acid was associated with higher relative abundance of Prevotellaceae.id.960 and lower relative abundance of Clostridiaceae1.id.1869 and Verrucomicrobiaceae.id.4036. In addition, genetic liability to the prescription of salicylic acid was associated with lower abundance of order Verrucomicrobiales.id.4030, class Verrucomicrobiae.id.4029 and phylum Verrucomicrobia.id.3982. These results suggested that the use of salicylic acid for pain management might have a multilevel effect on changes in the gut microbiota. It is well known that previous observational studies have concluded that many analgesic drugs, including salicylic acid, pose a wide range of impacts on the gut microbiota. Surprisingly, a different result was obtained in our study. Limited evidence was found between three mainstream analgesic drugs and gut microbiota, except for salicylic acid. Only a very small number of bacteria such as Eubacteriumxylanophilumgroup.id.14375 were restricted to the genus level by NSAID drugs and eventually proved to be significant. Similar results were registered for the effects of anilide and opioid on gut microbiota.

Causal relationships were also identified between the use of analgesics and metabolites in this study. Potential causal associations between salicylic acid use and four circulating metabolites including acetoacetate, creatinine, omega-3 fatty acids and triglycerides in large HDL. Notably, prior studies have indicated the functional roles of these metabolites in glucose uptake in the brain, memory decay and the development of Alzheimer’s disease [39,42]. Some potential causal links between anilides and opioids and intestinal microbial metabolites were also found in our findings, which were believed to potentially influence the metabolism of citrate, urea and glutamine. Likewise, the use of opioids could be associated with alterations in glucose and apolipoprotein A1 metabolism. Remarkably, some other studies have highlighted increased areas of urea in the brain that would likely represent a risk for Parkinson’s onset [43,44]. Altered glutamine metabolism had also been implicated as a link in the pathogenic mechanism of acute colitis, as well as decreased levels of apolipoprotein A1 in the cerebrospinal fluid predicting an increased risk of schizophrenia [45,46]. Considering the impact of common painkillers on the gut microbiota’s composition and metabolites, these findings present intriguing avenues to further investigate the link between painkiller use and neurological-related disorders mediated through the brain–gut axis.

### Strengths and limitations

This study has several advantages over the traditional observational studies. Firstly, we thus far for the first time applied the two-sample MR design to assess the causal relationship between analgesic drugs and gut microbiota. Secondly, the aggregated data on gut microbiota are the most integrated GWAS to date, and there are no overlapping samples in either exposure or outcome, which might avoid bias due to the winner’s curse phenomenon. Third, the IVW method was chosen as the primary approach due to its high efficiency in generating accurate MR estimates by assuming no pleiotropy among instrumental variables [26]. To address the issue of pleiotropy, the MR-Egger and MRPRESSO methods were employed, providing robust evidence of the MR results [47]. Both leave-one-out and Cochrane’s *Q* tests were utilized to identify heterogeneity [48]. Additionally, the directionality test was also applied to exclude reverse causality. The consistent direction of MR estimates increases our confidence in interpreting the results.

Some limitations remained to be noted in the presentation of our findings. First, although we utilized the largest GWAS on analgesic and gut microbiota, the limited number of SNPs found to be consistent with genome-wide significance might lead to weak genetic instrumentation. To address this issue, we relaxed the inclusion threshold for analgesic (*P*<5×10^−6^) and for gut microbiota (*P*<1×10^−5^) to include additional SNPs. Thereafter, we quantified the strength of genetic variation by calculating the F-statistic (>10) to identify SNPs that were included in further analyses. Second, the presence of horizontal pleiotropy was not detected in our study by applying the MRPRESSO and MR-Egger methods. Third, alterations in the gut microbiota were influenced by many factors, including diet, place of birth and lifestyle habits. These confounding factors were not present in the current study. Further studies are also needed to validate our results with relevant GWAS statistics in future work. Fourth, our MR studies were conducted based on the assumption of linear correlation, so the existence of a nonlinear relationship between exposure and outcome could not be completely excluded. Finally, the complete exclusion of indirect causal pathways was a challenge for all MR analyses and required us to utilize additional statistical methods to enhance the validity of the results in subsequent work.

In summary, the MR analysis approach was performed, thus revealing a potential causal relationship between the application of four common analgesics and gut microbiota composition as well as the circulating metabolites. Among them, salicylic drugs were more prominent. These findings may provide new research directions and evidence for the regulated use of clinical analgesics and the use of the brain–gut axis to intervene in some neuropsychiatric disorders.

## Supplementary material

10.1099/jmm.0.002028 Uncited Supplementary Material 1.

## Abbreviations

CIs: confidence intervals
CNS: central nervous system
FDR: false discovery rate
GI: gastrointestinal
GWASs: genome-wide association studies
HDL: high-density lipoprotein
IVW: inverse-variance weighted
LD: linkage disequilibrium
MR: Mendelian randomization
SNPs: single nucleotide polymorphisms
