# Supplementary material for: Assessing the impact of common pain medications on gut microbiota composition and metabolites: insights from a Mendelian randomization study
Source: J Med Microbiol. 2025 Jun 12;74(6):002028. doi: 10.1099/jmm.0.002028 (PMC12163180; doi:10.1099/jmm.0.002028)

Supplementary Materials for

This file includes:

Table S1. Basic information about the four pain medications studied studied and their active ingredients by ATC Category in UK Biobank.

Table S2. Summary of instrumental variables the for four pain medications studied.

Table S3. MR Power calculation for detecting significant ($P < 0.05$) causal effect of the four pain medications studied on the risk of gut microbiota and circulating metabolites.

Table S4. MR Results NSAID and Salicylic acid of on gut microbiota.

Table S5. MR Results the four pain medications studied of on circulating metabolites.

Figure S1. Scatterplot of Mendelian randomization (MR) estimates between NSAID, Salicylic acid and gut microbiota.

Figure S2. Scatterplot of Mendelian randomization (MR) estimates between the four pain medications studied and circulating metabolites.

Figure S3. Leave-one-out analysis of inverse-variance weighted (IVW) estimates between NSAID, Salicylic acid use, and genetically determined gut microbiota traits ($p < 1 \times 10^{-5}$).

Figure S4. Leave-one-out analysis of inverse-variance weighted (IVW) estimates between the four pain medications studied and genetically determined circulating metabolites traits ($p < 1 \times 10^{-5}$).

Table S1. Basic information about the four pain medications studied and their active ingredients by ATC Category in UK Biobank.

Category	Medication	UK Biobank Code	ATC Code	Drug Name/ Active Ingredient(s)
SALICYLIC ACID	aspro clear maximum strength soluble tablet	1140909480	N02BA01	Acetylsalicylic Acid
SALICYLIC ACID	disprin direct dispersible tablet	1140882192	N02BA01	Acetylsalicylic Acid
SALICYLIC ACID	anadin tablet	1140911754	N02BA51	Caffeine |Acetylsalicylic Acid
SALICYLIC ACID	beechams powder	1140911760	N02BA51	Caffeine |Acetylsalicylic Acid
SALICYLIC ACID	alka-seltzer tablet	1140917114	N02BA51	Acetylsalicylic Acid |Sodium Bicarbonate |Citric Acid
SALICYLIC ACID	migramax sachet powder	1141175756	N02BA51	Acetylsalicylic Acid |Metoclopramide
NSAID	indocid 25mg capsule	1140871354	M01AB01	Indomethacin
NSAID	sulindac	1140871604	M01AB02	Sulindac
NSAID	dicloflex 25mg e/c tablet	1140921828	M01AB05	Diclofenac
NSAID	voltarol 25mg e/c tablet	1140871168	M01AB05	Diclofenac
NSAID	diclomax sr 75mg m/r capsule	1140917394	M01AB05	Diclofenac
NSAID	rhumalgan 25mg e/c tablet	1140871180	M01AB05	Diclofenac
NSAID	volsaid retard 75mg m/r tablet	1140923920	M01AB05	Diclofenac
NSAID	motifene 75mg e/c+m/r capsule	1140909354	M01AB05	Diclofenac
NSAID	voltaren retard 100mg m/r tablet	1140877872	M01AB05	Diclofenac
NSAID	fenactol 25mg e/c tablet	1141182674	M01AB05	Diclofenac
NSAID	voltarol 100mg suppository	1140871174	M01AB05	Diclofenac
NSAID	rheumatac retard 75mg m/r tablet	1141167426	M01AB05	Diclofenac
NSAID	voltarene lp 100mg m/r tablet	1140877874	M01AB05	Diclofenac
NSAID	etodolac	1140871188	M01AB08	Etodolac
NSAID	lodine 200mg tablet	1140871196	M01AB08	Etodolac
NSAID	eccoxolac 300mg capsule	1141193170	M01AB08	Etodolac
NSAID	acemetacin	1140875278	M01AB11	Acemetacin
NSAID	ketorolac	1140884558	M01AB15 |S01BC05	Ketorolac
NSAID	aceclofenac	1140925806	M01AB16 |M02AA25	Aceclofenac
NSAID	arthrotec tablet	1140871266	M01AB55	Diclofenac |Misoprostol
NSAID	arthrotec 50 tablet	1140927086	M01AB55	Diclofenac |Misoprostol
NSAID	diclofenac sodium+misoprostol	1140878036	M01AB55	Diclofenac |Misoprostol
NSAID	feldene 10mg capsule	1140871672	M01AC01	Piroxicam
NSAID	brexidol 20mg tablet	1141169530	M01AC01	Piroxicam
NSAID	piroxicam	1140871666	M01AC01 |M02AA07 |S01BC06	Piroxicam
NSAID	tenoxicam	1140875346	M01AC02	Tenoxicam
NSAID	meloxicam	1140926732	M01AC06	Meloxicam
NSAID	mobic 15mg tablet	1140926796	M01AC06	Meloxicam
NSAID	mobic 7.5mg tablet	1140926794	M01AC06	Meloxicam
NSAID	nurofen 200mg tablet	1141187776	M01AE01	Ibuprofen
NSAID	cuprofen 200mg tablet	1140871388	M01AE01	Ibuprofen
NSAID	anadin ibuprofen 200mg tablet	1141153134	M01AE01	Ibuprofen
NSAID	naprosyn 250mg tablet	1140871472	M01AE02	Naproxen
NSAID	oruvail 100 m/r capsule	1140871522	M01AE03	Ketoprofen
NSAID	ketoprofen	1140871506	M01AE03 |M02AA10	Ketoprofen
NSAID	froben 50mg tablet	1140871238	M01AE09	Flurbiprofen
NSAID	flurbiprofen	1140871236	M01AE09 |M02AA19 |R02AX01 |S01BC04	Flurbiprofen
NSAID	surgam 200mg tablet	1140871616	M01AE11	Tiaprofenic Acid
NSAID	ibuprofen+menthol 5%/3% gel	1140911748	M01AE51	Ibuprofen |Menthol
NSAID	napratec tablet combination pack	1140871638	M01AE56	Naproxen |Misoprostol
NSAID	naproxen+misoprostol	1140881612	M01AE56	Naproxen |Misoprostol
NSAID	mefenamic acid	1140871542	M01AG01	Mefenamic Acid

NSAID	ponstan 250mg capsule	1140871546	M01AG01	Mefenamic Acid
NSAID	tolfenamic acid	1140928840	M01AG02	Tolfenamic Acid
NSAID	celebrex 100mg capsule	1141176668	M01AH01	Celecoxib
NSAID	celebrex 200mg capsule	1141176670	M01AH01	Celecoxib
NSAID	etoricoxib	1141180140	M01AH05	Etoricoxib
NSAID	arcoxia 60mg tablet	1141180148	M01AH05	Etoricoxib
NSAID	arcoxia 90mg tablet	1141180150	M01AH05	Etoricoxib
NSAID	arcoxia 120mg tablet	1141180152	M01AH05	Etoricoxib
NSAID	nabumetone	1140875336	M01AX01	Nabumetone
NSAID	relifex 500mg tablet	1140875338	M01AX01	Nabumetone
NSAID	glucosamine product	1141188442	M01AX05	Glucosamine
NSAID	chondroitin product	1187	M01AX25	Chondroitin Sulfate
OPIOIDS	tylex capsule	1140871680	N02AJ06	Acetaminophen |Codeine
OPIOIDS	zapain caplet	1141178052	N02AJ06	Acetaminophen |Codeine
OPIOIDS	solpadeine capsule	1141168650	N02AJ06	Caffeine |Acetaminophen |Codeine
OPIOIDS	kapake tablet	1140864070	N02AJ06	Acetaminophen |Codeine
OPIOIDS	codipar caplet	1141187304	N02AJ06	Acetaminophen |Codeine
OPIOIDS	solpadeine tablet	1141168648	N02AJ06	Acetaminophen |Codeine
OPIOIDS	kapake capsule	1141165512	N02AJ06	Acetaminophen |Codeine
OPIOIDS	solpadol effervescent tablet	1140871682	N02AJ06	Acetaminophen |Codeine
OPIOIDS	zapain capsule	1141178054	N02AJ06	Acetaminophen |Codeine
OPIOIDS	paracodol capsule	1140925778	N02AJ06	Acetaminophen |Codeine
OPIOIDS	migraleve yellow tablet	1140872030	N02AJ06	Acetaminophen |Codeine
OPIOIDS	kapake 30/500 effervescent tablet	1141190656	N02AJ06	Acetaminophen |Codeine
OPIOIDS	solpadeine forte dispersible tablet	1140856340	N02AJ06	Caffeine |Acetaminophen |Codeine
OPIOIDS	codis dispersible tablet	1140856336	N02AJ07	Codeine |Acetylsalicylic Acid
OPIOIDS	aspirin+codeine	1140882392	N02AJ07	Codeine |Acetylsalicylic Acid
OPIOIDS	co-codaprin	1140923344	N02AJ07	Codeine |Acetylsalicylic Acid
OPIOIDS	ibuprofen+codeine phosphate	1140878030	N02AJ08	Codeine |Ibuprofen
OPIOIDS	cuprofen plus tablet	1141190952	N02AJ08	Codeine |Ibuprofen
OPIOIDS	migraleve tablet	1141168554	N02AJ09	Acetaminophen |Codeine |Buclizine
OPIOIDS	solpadeine soluble effervescent tablet	1140856442	N02AJ09	Caffeine |Acetaminophen |Codeine
OPIOIDS	solpadeine plus soluble effervescent tablet	1141189064	N02AJ09	Caffeine |Acetaminophen |Codeine
OPIOIDS	solpadeine plus capsule	1141189008	N02AJ09	Caffeine |Acetaminophen |Codeine
OPIOIDS	solpadeine max tablet	1141167748	N02AJ09	Acetaminophen |Codeine
OPIOIDS	solpadeine plus tablet	1141189010	N02AJ09	Caffeine |Acetaminophen |Codeine
OPIOIDS	veganin tablet	1140856348	N02AJ09	Caffeine |Acetaminophen |Codeine
OPIOIDS	propain tablet	1140856436	N02AJ09	Caffeine |Acetaminophen |Codeine |Diphenhydramine
OPIOIDS	propain caplet	1141172966	N02AJ09	Caffeine |Acetaminophen |Codeine |Diphenhydramine
OPIOIDS	tramacet 325mg/37.5mg tablet	1141190960	N02AJ13	Tramadol |Acetaminophen
OPIOIDS	tramadol	1140864992	N02AX02	Tramadol
OPIOIDS	zydol 50mg capsule	1140865000	N02AX02	Tramadol
OPIOIDS	zamadol 50mg capsule	1140928742	N02AX02	Tramadol
OPIOIDS	zamadol sr 100mg m/r capsule	1141153424	N02AX02	Tramadol
OPIOIDS	zydol sr 100mg m/r tablet	1140922636	N02AX02	Tramadol
OPIOIDS	zydol soluble 50mg tablet	1140928372	N02AX02	Morphine
OPIOIDS	meptazinol	1140881026	N02AX05	Meptazinol Hydrochloride
OPIOIDS	meptid 200mg tablet	1140881028	N02AX05	Meptazinol Hydrochloride
OPIOIDS	paracetamol+tramadol	1141190956	N02AX52	Tramadol |Acetaminophen
ANILIDES	paracetamol	2038460150	N02BE01	Acetaminophen

ANILIDES	paracetamol product	1140868240	N02BE01	Acetaminophen
ANILIDES	anadin paracetamol 500mg tablet	1141188516	N02BE01	Acetaminophen
ANILIDES	panadol 500mg tablet	1140856240	N02BE01	Acetaminophen
ANILIDES	panadol 500mg capsule	1140856238	N02BE01	Acetaminophen
ANILIDES	hedex tablet	1140911758	N02BE01	Acetaminophen
ANILIDES	paramax tablet	1140872036	N02BE51	Acetaminophen |Metoclopramide
ANILIDES	solpadeine headache soluble tablet	1141189068	N02BE51	Caffeine |Acetaminophen
ANILIDES	non-drowsy sinutab tablet	1141188512	N02BE51	Acetaminophen |Pseudoephedrine
ANILIDES	sudafed dual relief capsule	1141171672	N02BE51	Acetaminophen |Phenylephrine
ANILIDES	lemsip sachet	1140911762	N02BE51	Acetaminophen |Phenylephrine
ANILIDES	panadol extra tablet	1141168646	N02BE51	Caffeine |Acetaminophen
ANILIDES	solpadeine headache tablet	1141189066	N02BE51	Caffeine |Acetaminophen
ANILIDES	paracetamol+caffeine	1140884404	N02BE51	Caffeine |Acetaminophen
ANILIDES	midrid capsule	1140871966	N02BE51	Acetaminophen |Isometheptene
ANILIDES	paracetamol+metoclopramide 500mg/5mg tablet	1140872044	N02BE51	Acetaminophen |Metoclopramide

NSAIDS, Anti-inflammatory and antirheumatic products, non-steroids; SALICYLIC ACID, Salicylic acid and derivatives; ATC, Anatomical Therapeutic Chemical Classification System.

Table S2. Summary of instrumental variables for the four pain medications studied.

Analgesics	SNP	CHR	POS (hg 19)	GENE	EA	OA	EAF	Beta	se	P-value	R2	F
	rs12029331	1	154160885	TPM3	T	C	0.42	0.0322	0.0066	1.30E-06	0.00013	23
	rs2274319	1	156450873	MEF2D	T	C	0.35	0.036	0.0069	1.50E-07	0.00015	28
	rs12568655	1	174434332	RABGAP1L	A	G	0.39	-0.0407	0.0067	1.20E-09	0.00021	37
	rs2430157	1	187819353	RP5-925F19.1	T	C	0.47	-0.0308	0.0065	2.60E-06	0.00012	22
	rs41272663	2	211302627	LANCL1	A	C	0.26	-0.035	0.0074	2.40E-06	0.00012	22
	rs62180637	2	217424243	RPL37A	C	T	0.47	-0.0313	0.0066	1.80E-06	0.00013	23
	rs4663983	2	234815005	STK25	G	A	0.19	-0.0558	0.0083	1.70E-11	0.00025	45
	rs7567892	2	242470476	BOK-AS1	T	C	0.1	0.0614	0.011	2.00E-08	0.00017	31
	rs59357103	3	49890967	TRAIP	A	G	0.17	0.041	0.0088	3.30E-06	0.00012	22
	rs10033431	4	67782113	AC112518.3	C	T	0.27	0.0376	0.0074	4.10E-07	0.00014	26
	rs17449582	4	87240157	MAPK10	T	C	0.36	0.0342	0.0068	4.20E-07	0.00014	26
	rs4356969	4	112302592	RP11-119H12.3	C	G	0.26	-0.0363	0.0075	1.40E-06	0.00013	23
	rs62378567	5	173106225	HMP19	G	A	0.02	-0.1156	0.0237	1.00E-06	0.00013	24
	rs11242905	6	474405	RP11-157J24.2	C	T	0.13	0.0452	0.0098	4.30E-06	0.00012	21
	rs17191307	6	31178371	TBC1D22B	G	T	0.25	0.0355	0.0075	2.20E-06	0.00012	22
	rs3130486	6	31722780	MSH5	T	C	0.27	-0.0549	0.0074	1.00E-13	0.00031	55
	rs6568392	6	96861476	UFL1-AS1	G	T	0.22	0.0402	0.0079	3.60E-07	0.00014	26
	rs4718618	7	67224778	RP11-421N10.1	A	G	0.19	0.0395	0.0084	2.70E-06	0.00012	22
Anilides	rs73168813	7	135846513	AC009784.3	C	G	0.02	0.1063	0.0224	2.10E-06	0.00013	22
	rs4741328	9	13764507	LINC00583	C	T	0.37	-0.033	0.0068	1.10E-06	0.00013	24
	rs73531210	9	112519447	PALM2	T	C	0.05	-0.0699	0.0149	2.50E-06	0.00012	22
	rs1648368	11	115896500	AP000797.2	T	C	0.13	-0.0467	0.0098	2.00E-06	0.00013	23
	rs11172113	12	57527283	LRP1	C	T	0.41	-0.0534	0.0066	8.90E-16	0.00036	65
	rs1544115	13	59215386	DNAJA1P1	T	C	0.25	0.035	0.0076	4.30E-06	0.00012	21
	rs9571576	13	66936133	PCDH9	C	T	0.49	0.0318	0.0066	1.20E-06	0.00013	24
	rs2526922	14	73101118	DPF3	A	G	0.47	-0.0304	0.0065	3.50E-06	0.00012	22
	rs75036981	14	93463880	ITPK1	A	G	0.06	0.0659	0.0137	1.50E-06	0.00013	23
	rs12438004	15	46316832	RP11-315O8.1	C	A	0.42	0.0352	0.0067	1.30E-07	0.00015	28
	rs2930291	15	74604834	CCDC33	A	G	0.36	-0.032	0.0068	2.60E-06	0.00012	22
	rs9925184	16	5800933	RP11-420N3.3	C	T	0.44	0.0328	0.0067	8.10E-07	0.00014	24
	rs74618578	16	54646068	AC007491.1	G	A	0.03	-0.1037	0.0206	4.60E-07	0.00014	25
	rs9915717	17	10561049	MYH3	A	G	0.2	0.0417	0.0082	3.10E-07	0.00015	26
	rs17652520	17	44098967	MAPT	A	G	0.23	0.0557	0.0078	8.10E-13	0.00028	51
	rs73998427	17	70840296	SLC39A11	T	C	0.03	0.0875	0.0187	2.90E-06	0.00012	22
	rs1942262	18	52873317	TCF4	A	G	0.29	0.0369	0.0072	3.00E-07	0.00015	26
	rs293566	20	31097877	NOL4L	C	T	0.33	0.0347	0.0069	5.70E-07	0.00014	25
	rs2825128	21	20172863	AP000472.3	C	A	0.45	0.0314	0.0066	1.90E-06	0.00013	23
	rs4110942	1	29337802	EPB41	G	A	0.09	-0.0564	0.0122	3.90E-06	0.00013	21
	rs7532754	1	115824515	NGF	T	C	0.36	-0.0343	0.0072	1.70E-06	0.00014	23
	rs3737240	1	150483355	ECM1	T	C	0.39	-0.0368	0.007	1.80E-07	0.00017	27
	rs1632360	1	154267672	RNU6-239P	G	A	0.06	0.0789	0.0152	2.00E-07	0.00016	27
	rs12409445	1	192403191	AL136987.1	C	T	0.19	-0.0448	0.009	6.80E-07	0.00015	25
	rs3821269	2	70698590	TGFA	A	G	0.5	0.0375	0.0069	5.90E-08	0.00018	29
	rs7687973	4	81139104	RP11-576N17.4	C	T	0.22	-0.0443	0.0083	1.00E-07	0.00017	28
	rs11097493	4	97020614	MANBA	A	T	0.36	0.034	0.0072	2.30E-06	0.00014	22
	rs151415	4	103137539	MANBA	G	C	0.24	0.0426	0.0082	2.10E-07	0.00016	27
	rs56166763	4	140943169	MAML3	C	G	0.37	-0.0409	0.0072	1.20E-08	0.0002	33
	rs12522598	5	120174466	LMNB1	G	A	0.29	0.0452	0.0076	3.00E-09	0.00021	35
	rs6891880	5	149972794	CTB-95D12.1	G	A	0.43	-0.0385	0.0071	4.90E-08	0.00018	30
	rs359431	5	173288534	HMP19	C	T	0.44	0.0367	0.007	1.30E-07	0.00017	28
	rs2517611	6	30169327	TRIM26	G	A	0.23	-0.0505	0.0082	7.10E-10	0.00023	38
	rs114212906	6	31391389	HCP5	T	C	0.05	0.0921	0.0163	1.70E-08	0.00019	32
	rs2237257	6	112005126	FYN	G	T	0.12	-0.0515	0.0106	1.10E-06	0.00014	24
	rs1589738	6	147967761	SAMD5	A	G	0.37	0.0332	0.0072	3.60E-06	0.00013	21
	rs11993835	8	88867707	CTB-118P15.3	A	G	0.13	-0.0489	0.0102	1.70E-06	0.00014	23
	rs2608029	8	129170126	MIR1208	G	C	0.33	0.0362	0.0073	8.50E-07	0.00015	24
NSAID	rs71510292	9	91898790	CKS2	C	T	0.05	0.0817	0.0163	5.40E-07	0.00015	25
	rs7856204	9	95173814	CENPP	C	G	0.36	0.0344	0.0072	1.80E-06	0.00014	23
	rs55842862	9	119275331	ASTN2	C	G	0.07	0.0629	0.0131	1.60E-06	0.00014	23
	rs113041162	10	71106964	HK1	A	G	0.15	0.0499	0.0098	3.20E-07	0.00016	26
	rs11597763	10	98244903	TLL2	G	A	0.2	0.043	0.0086	5.30E-07	0.00015	25
	rs1544861	11	10679441	MRVI1	T	C	0.33	-0.0366	0.0074	6.60E-07	0.00015	25
	rs12285912	11	126727315	KIRREL3	G	A	0.03	0.1033	0.0216	1.70E-06	0.00014	23
	rs11171710	12	56368078	RAB5B	A	G	0.45	0.0339	0.007	1.40E-06	0.00014	23
	rs3001426	12	57509055	STAT6	C	T	0.45	-0.0562	0.007	1.00E-15	0.00039	64
	rs1458612	12	75483802	KCNC2	T	C	0.3	-0.0348	0.0075	3.70E-06	0.00013	21
	rs17081175	13	66919700	PCDH9	A	C	0.26	-0.0381	0.0079	1.30E-06	0.00014	23
	rs8013531	14	23247687	SLC7A7	T	C	0.41	0.034	0.0071	1.40E-06	0.00014	23
	rs56044130	14	94821812	SERPINA2	G	A	0.09	0.058	0.0118	9.40E-07	0.00015	24
	rs533650768	15	74357425	GOLGA6A	G	A	0.02	0.1263	0.0262	1.50E-06	0.00014	23
	rs34862454	15	75101530	LMAN1L	C	T	0.33	-0.0402	0.0073	4.40E-08	0.00018	30
	rs56236914	17	43483551	ARHGAP27	T	C	0.18	0.0486	0.0089	5.50E-08	0.00018	30
	rs55938136	17	43798360	LINC02210-CRHR1	G	A	0.23	0.0417	0.0083	4.70E-07	0.00015	25
	rs10164103	18	65419274	RP11-638L3.1	G	A	0.1	-0.0576	0.0117	8.00E-07	0.00015	24
	rs144713500	19	5158138	KDM4B	A	G	0.01	0.1401	0.0306	4.50E-06	0.00013	21
Opioids	rs945211	1	32191798	ADGRB2	G	C	0.38	-0.0514	0.011	3.2E-06	0.00028	22

	rs2618039	1	112324111	KCND3	T	A	0.38	0.0612	0.0111	3.2E-08	0.00039	31
	rs138200696	1	117727725	VTCN1	C	A	0.02	0.1988	0.0424	2.8E-06	0.00028	22
	rs139152977	2	199098906	PLCL1	A	T	0.03	0.1638	0.0344	1.9E-06	0.00029	23
	rs138405014	2	221947203	SERPINE2	A	G	0.01	-0.2178	0.0475	4.6E-06	0.00027	21
	rs140729888	3	10521537	ATP2B2	C	T	0.01	-0.205	0.0446	4.3E-06	0.00027	21
	rs7428430	3	50174184	SEMA3F-AS1	T	C	0.48	-0.0652	0.0107	1.20E-09	0.00047	37
	rs7631941	3	80480052	LINC02050	T	C	0.13	0.073	0.016	4.9E-06	0.00026	21
	rs521956	3	173117975	NLGN1	T	C	0.48	-0.0538	0.0107	5.1E-07	0.00032	25
	rs76034781	4	3272782	MSANTD1	A	G	0.08	0.0981	0.0202	1.2E-06	0.0003	24
	rs13135092	4	103198082	SLC39A8	G	A	0.08	0.0987	0.0197	5.2E-07	0.00032	25
	rs1397440	4	140892872	MAML3	T	C	0.33	-0.0535	0.0115	3.5E-06	0.00027	22
	rs11743674	5	151418837	LINC01933	C	G	0.22	0.0605	0.0129	2.6E-06	0.00028	22
	rs2253491	6	31249217	HLA-B	A	G	0.21	-0.067	0.0132	3.6E-07	0.00033	26
	rs56226325	7	2078981	MAD1L1	T	C	0.15	-0.0745	0.0148	4.9E-07	0.00032	25
	rs117710046	8	59430010	RP11-114M5.3	T	C	0.02	0.2097	0.0431	1.2E-06	0.0003	24
	rs1519369	8	139393909	FAM135B	T	C	0.33	-0.0533	0.0116	4E-06	0.00027	21
	rs17487601	9	37213641	ZCCHC7	A	G	0.36	-0.0555	0.0112	7.7E-07	0.00031	24
	rs7860752	9	81389671	MTND2P8	G	A	0.19	0.0695	0.0136	3.4E-07	0.00033	26
	rs12238134	9	128746612	PBX3	A	G	0.31	-0.0715	0.0116	8.20E-10	0.00048	38
	rs12357321	10	21790476	CASC10	A	G	0.31	0.0632	0.0117	7.1E-08	0.00037	29
	rs4258296	10	134953361	RPL5P28	C	T	0.36	0.0555	0.0112	6.5E-07	0.00031	25
	rs7110825	11	46120317	PHF21A	C	T	0.27	0.057	0.0121	2.3E-06	0.00028	22
	rs4763505	12	10477712	KLRD1	T	A	0.32	-0.0531	0.0116	4.5E-06	0.00027	21
	rs56262049	13	42644705	DGKH	C	T	0.18	-0.0663	0.0141	2.5E-06	0.00028	22
	rs7319102	13	58252801	PCDH17	G	A	0.23	0.0603	0.0128	2.4E-06	0.00028	22
	rs7154623	14	73798243	NUMB	G	A	0.19	0.0644	0.0136	2.4E-06	0.00028	22
	rs2289739	15	41801512	LTK	T	G	0.34	0.0543	0.0114	2E-06	0.00029	23
	rs75169822	15	75887405	SNUPN	G	A	0.26	0.0592	0.0122	1.3E-06	0.0003	23
	rs113544339	15	86576076	LINC01584	G	T	0.02	-0.1657	0.0358	3.6E-06	0.00027	21
	rs975376	16	73100503	RP11-346C20.3	C	G	0.16	0.0712	0.0146	1E-06	0.0003	24
	rs59695806	17	43156023	NMT1	C	T	0.29	0.0574	0.0118	1.1E-06	0.0003	24
	rs6141277	20	30831157	POFUT1	C	G	0.39	0.0521	0.011	2.2E-06	0.00028	22
	rs2297197	20	44673546	SLC12A5	G	C	0.28	0.0602	0.0122	7.7E-07	0.00031	24
	rs73228014	21	42752754	MX2	A	G	0.02	0.161	0.0348	3.8E-06	0.00027	21
Salicylic Acid	rs11206268	1	54445348	HNRNPA3P12	G	A	0.07	-0.0754	0.0159	2.2E-06	0.0002	22
	rs583104	1	109821307	CELSR2	G	T	0.22	-0.078	0.0095	1.70E-16	0.00061	68
	rs10218528	1	174447588	RABGAP1L	A	T	0.39	-0.0411	0.0082	4.5E-07	0.00023	25
	rs113242618	1	234118046	SLC35F3	A	G	0.01	-0.1542	0.0332	3.4E-06	0.00019	22
	rs896232	2	2732877	AC018685.2	T	C	0.36	-0.0395	0.0082	1.5E-06	0.00021	23
	rs4299376	2	44072576	-	G	T	0.32	0.0453	0.0085	8.6E-08	0.00026	29
	rs9865841	3	135876125	MSL2	A	G	0.22	-0.0451	0.0096	2.3E-06	0.0002	22
	rs13082582	3	170202670	SLC7A14	C	G	0.4	0.0391	0.008	1.2E-06	0.00021	24
	rs1825548	4	20150967	MTCO3P44	T	A	0.12	0.0553	0.012	3.9E-06	0.00019	21
	rs12188010	5	166996466	TENM2	A	T	0.38	0.0398	0.0082	1.2E-06	0.00021	24
	rs2523589	6	31327334	HLA-B	T	G	0.5	0.0444	0.0079	2.1E-08	0.00028	31
	rs1931656	6	82610188	RP11-379B8.1	A	T	0.46	0.0369	0.008	4E-06	0.00019	21
	rs117733303	6	160922870	LPAL2	G	A	0.02	0.1846	0.0285	8.90E-11	0.00038	42
	rs74617384	6	160997118	LPA	T	A	0.08	0.105	0.0144	2.70E-13	0.00048	53
	rs56226325	7	2078981	MAD1L1	T	C	0.15	-0.0567	0.0109	2.2E-07	0.00024	27
	rs75278536	8	19821425	LPL	G	T	0.11	-0.0673	0.0128	1.6E-07	0.00024	27
	rs75786943	8	97103955	RP11-44N17.1	C	G	0.02	0.149	0.0311	1.6E-06	0.00021	23
	rs28601761	8	126500031	RP11-136O12.2	G	C	0.42	-0.0607	0.0082	1.30E-13	0.00049	55
	rs1831733	9	22076071	CDKN2B-AS1	C	T	0.48	0.0458	0.008	9.40E-09	0.00029	33
	rs635634	9	136155000	ABO	T	C	0.18	0.0666	0.0102	7.40E-11	0.00038	42
	rs10508267	10	3846959	KLF6	C	G	0.36	-0.0393	0.0084	2.7E-06	0.0002	22
	rs10887793	10	89990905	RP11-129G17.2	T	G	0.45	0.0427	0.008	1E-07	0.00025	28
	rs77507211	11	24918930	LUZP2	T	G	0.02	0.1365	0.0299	4.8E-06	0.00019	21
	rs59681006	11	70179401	PPFIA1	C	T	0.13	-0.0566	0.0118	1.7E-06	0.0002	23
	rs964184	11	116648917	ZPR1	G	C	0.13	0.0633	0.0116	4.9E-08	0.00027	30
	rs113551213	12	12513870	BORCS5	A	G	0.05	0.0851	0.0185	4.1E-06	0.00019	21
	rs7315004	12	31928182	H3F3C	A	T	0.42	-0.0408	0.0081	4.6E-07	0.00023	25
	rs12580718	12	67407656	RP11-123O10.4	A	G	0.37	-0.0391	0.0083	2.2E-06	0.0002	22
	rs1960743	13	83832609	RNU6-67P	A	G	0.11	0.0586	0.0127	4E-06	0.00019	21
	rs4995008	13	106831286	RNA5SP38	A	G	0.13	0.0541	0.0118	4.3E-06	0.00019	21
	rs950100	14	42971842	RP11-214N1.1	A	G	0.07	-0.0715	0.0156	4.5E-06	0.00019	21
	rs185197153	16	58450949	LINC02137	C	A	0.01	-0.1632	0.0347	2.6E-06	0.0002	22
	rs11874619	18	46500936	RP11-1058N17.1	C	G	0.39	-0.0386	0.0083	3.3E-06	0.00019	22
	rs7244655	18	67986087	SOCS6	A	G	0.25	-0.0433	0.0092	2.2E-06	0.0002	22
	rs73015016	19	11191300	LDLR	A	G	0.12	-0.0957	0.0123	6.40E-15	0.00054	61
	rs7412	19	45412079	APOE	T	C	0.08	-0.1108	0.0147	4.90E-14	0.00051	57
	rs117571676	20	21002369	APMAP	A	T	0.02	-0.1332	0.0285	3.1E-06	0.00019	22
	rs9982261	21	17528955	MIR99AHG	A	G	0.01	-0.1749	0.0376	3.3E-06	0.00019	22
	rs9612563	22	24597540	MYO18B	C	A	0.06	-0.0799	0.0171	2.8E-06	0.0002	22
	rs139696858	22	47836888	-	A	G	0.01	-0.1642	0.0342	1.6E-06	0.00021	23

Table S3. MR Power calculation for detecting significant (P < 0.05) causal effect of the four pain medications studied on the risk of gut microbiota and circulating metabolites.

			Outcome			
			gut microbiota		metabolites	
Exposure	NSNPs	R^2	Power (beta=0.2)	Power (beta=0.3)	Power (beta=0.2)	Power (beta=0.3)
NSAID	38	0.006	55.5%	88.2%	68.6%	95.6%
Salicylic acid	40	0.010	77.3%	98.2%	88.4%	99.7%
Anilides	37	0.006	55.5%	88.2%	68.6%	95.6%
Opioids	35	0.010	77.3%	98.2%	88.4%	99.7%

MR, mendelian randomization; NSNPs, number of single nucleotide polymorphisms; R^2, the proportion of variance explained by exposure. R^2 for each SNP: R^2 = [beta. exposure^2]/ [se. exposure^2 * N + beta. exposure^2]; R^2 combined= SUM[R^2].

Table S4. MR Results NSAID and Salicylic acid of on gut microbiota.

						Directional pleiotropy		Cochran Q-test		Steiger
Exposure	Outcome	Method	NSNPs	Beta (95%CI)	pval	Egger intercept (P)	MRPRESSO global test RSSobs (P)	I^2 statistics	Q-statistic (P)	P
NSAID	genus.Eubacteriumxylanophilumgroup	IVW	22	0.152 (0.080, 0.225)	0.035	−0.031 (0.044)	38.044 (0.365)	14%	28.323 (0.394)	65.8E-03
		MR Egger	22	0.207 (0.110, 0.303)	0.032					
		Weighted median	22	0.203 (0.050, 0.355)	0.198					
		Simple mode	22	0.198 (0.038, 0.358)	0.230					
		Weighted mode	22	0.140(−0.275, 0.555)	0.739					
Salicylic acid	family.Prevotellaceae.id.960	IVW	20	0.161 (0.102, 0.220)	0.006	−0.005 (0.683)	31.724 (0.525)	0%	10.006 (0.968)	9.57E-05
		MR Egger	20	0.094 (0.017, 0.170)	0.219					
		Weighted median	20	0.079 (−0.049, 0.206)	0.544					
		Simple mode	20	0.066 (−0.074, 0.206)	0.641					
		Weighted mode	20	0.220 (0.020, 0.421)	0.286					
	family.Clostridiaceae1.id.1869	IVW	21	−0.141 (−0.201, −0.081)	0.018	−0.003 (0.798)	29.933 (0.599)	0%	10.468 (0.959)	1.83E-07
		MR Egger	21	−0.116 (−0.197, −0.036)	0.149					
		Weighted median	21	−0.015 (−0.160, 0.133)	0.921					
		Simple mode	21	−0.265 (−0.416, −0115)	0.094					
		Weighted mode	21	−0.091 (−0.293, 0.111)	0.658					
	class.Verrucomicrobiae.id.4029	IVW	19	−0.175 (−0.243, −0.108)	0.009	0.014 (0.297)	34.996 (0.4)	0%	18.005 (0.587)	1.55E-04
		MR Egger	19	−0.186 (−0.274, −0.098)	0.035					
		Weighted median	19	−0.171 (−0.309, −0.033)	0.232					
		Simple mode	19	−0.199 (−0.350, −0.047)	0.207					
		Weighted mode	19	−0.263 (−0.487, −0.038)	0.257					
	family.Verrucomicrobiaceae.id.4036	IVW	19	−0.175 (−0.242, −0.107)	0.010	0.013 (0.3)	34.855 (0.411)	6%	17.907 (0.594)	1.48E-04
		MR Egger	19	−0.184 (−0.275, −0.093)	0.043					
		Weighted median	19	−0.173 (−0.304, −0.041)	0.205					
		Simple mode	19	−0.198 (−0.362, −0.034)	0.243					
		Weighted mode	19	−0.261 (−0.486, −0.037)	0.260					
	genus.Adlercreutzia.id.812	IVW	15	−0.177 (−0.264, −0.090)	0.043	0.014 (0.426)	39.952 (0.177)	17%	24.317 (0.184)	78.8E-03
		MR Egger	15	−0.265 (−0.382, −0.147)	0.025					
		Weighted median	15	−0.333 (−0.504, −0.162)	0.072					
		Simple mode	15	−0.314 (−0.508, −0.120)	0.128					
		Weighted mode	15	−0.649 (−0.925, −0.373)	0.035					
	genus.Akkermansia.id.4037	IVW	19	−0.174 (−0.241, −0.106)	0.010	0.014 (0.298)	34.732 (0.391)	0%	17.844 (0.598)	1.40E-04
		MR Egger	19	−0.185 (−0.277, −0.092)	0.045					
		Weighted median	19	−0.175 (−0.309, −0.042)	0.205					
		Simple mode	19	−0.205 (−0.356, −0.055)	0.190					
		Weighted mode	19	−0.261 (−0.485, −0.037)	0.261					
	genus.Clostridiumsensustricto1.id.1873	IVW	20	−0.125 (−0.186, −0.064)	0.040	−0.004 (0.755)	29.158 (0.642)	0%	11.023 (0.946)	2.06E-07
		MR Egger	20	−0.091 (−0.172, −0.010)	0.264					
		Weighted median	20	−0.0245 (−0.162, 0.113)	0.861					
		Simple mode	20	−0.244 (−0.382, −0.105)	0.095					
		Weighted mode	20	−0.020 (−0.227, 0.187)	0.923					
	order.Verrucomicrobiales.id.4030	IVW	19	−0.175 (−0.243, −0.108)	0.009	0.014 (0.297)	34.996 (0.362)	6%	18.005 (0.587)	1.55E-04
		MR Egger	19	−0.186 (−0.275, −0.097)	0.038					
		Weighted median	19	−0.171 (−0.311, −0.030)	0.239					
		Simple mode	19	−0.199 (−0.361, −0.036)	0.239					
		Weighted mode	19	−0.263 (−0.487,	0.257					

				−0.038)						
	phylum.Verrucomicrobia.id.3982	IVW	19	−0.142 (−0.209, −0.075)	0.033	0.011 (0.375)	36.328 (0.335)	6%	18.188 (0.575)	1.12E−04
		MR Egger	19	−0.156 (−0.244, −0.067)	0.079					
		Weighted median	19	−0.148 (−0.280, −0.016)	0.278					
		Simple mode	19	−0.239 (−0.395, −0.083)	0.142					
		Weighted mode	19	−0.304 (−0.520, −0.087)	0.179					
MR, mendelian randomization; NSAID and Salicylic acid, common pain medications; IVW, inverse−variance weighted; NSNPs, number of single nucleotide polymorphisms; Beta, mendelian randomization effect estimate; CI, confidence interval; RSSobs, residual sums of squares of observations;										

Table S5. MR Results the four pain medications studied of on circulating metabolites.

						Directional pleiotropy		Cochran Q-test		Steiger
Exposure	Outcome	Method	NSNPs	Beta (95%CI)	pval	Egger intercept (P)	MRPRESSO global test RSSobs (P)	I^2 statistics	Q-statistic (P)	P
Anilide	Citrate	Inverse variance weighted	32	−0.109 (−0.059, −0.159)	0.030	−0.003 (0.666)	30.314 (0.658)	0%	28.640 (0.637)	4.71E−07
		MR Egger	32	0.052 (−0.141, 0.246)	0.788					
		Weighted median	32	−0.148 (−0.217, −0.078)	0.034					
		Simple mode	32	−0.318 (−0.472, −0.164)	0.048					
		Weighted mode	32	−0.302 (−0.440, −0.164)	0.037					
	Glutamine	Inverse variance weighted	31	−0.102 (−0.050, −0.153)	0.047	0.00004 (0.996)	35.121 (0.429)	3%	32.791 (0.429)	5.43E−06
		MR Egger	31	−0.079 (−0.273, 0.116)	0.689					
		Weighted median	31	−0.135 (−0.204, −0.066)	0.051					
		Simple mode	31	−0.153 (−0.292, −0.013)	0.283					
		Weighted mode	31	−0.153 (−0.294, −0.012)	0.287					
	Urea	Inverse variance weighted	31	−0.124 (−0.181, −0.066)	0.031	−0.018 (0.045)	28.156 (0.662)	3%	30.162 (0.644)	1.48E−04
		MR Egger	31	0.086 (−0.154, 0.326)	0.723					
		Weighted median	31	−0.147 (−0.227, −0.067)	0.067					
		Simple mode	31	−0.150 (−0.304, 0.004)	0.339					
		Weighted mode	31	−0.140 (−0.281, 0.002)	0.331					
NSAID	Acetoacetate	Inverse variance weighted	27	−0.115 (−0.172, −0.059)	0.042	0.008 (0.386)	26.234 (0.714)	8%	24.705 (0.693)	4.29E−05
		MR Egger	27	−0.208 (−0.411, −0.004)	0.317					
		Weighted median	27	−0.096 (−0.171, −0.021)	0.199					
		Simple mode	27	−0.052 (−0.195, 0.090)	0.716					
		Weighted mode	27	−0.038 (−0.155, 0.079)	0.746					
Opioid	Apolipoprotein A−I	Inverse variance weighted	24	0.087 (0.046, 0.128)	0.033	−0.006 (0.471)	17.893 (0.856)	0%	16.428 (0.836)	1.60E−10
		MR Egger	24	0.170 (0.050, 0.290)	0.171					
		Weighted median	24	0.099 (0.042, 0.156)	0.084					
		Simple mode	24	0.124 (0.010, 0.238)	0.289					
		Weighted mode	24	0.113 (0.018, 0.207)	0.244					
	Glucose	Inverse variance weighted	26	0.073 (0.108, 0.038)	0.038	−0.002 (0.823)	15.762 (0.960)	0%	14.633 (0.950)	3.27E−17
		MR Egger	26	0.096 (−0.012, 0.204)	0.382					
		Weighted median	26	0.084 (0.036, 0.132)	0.079					
		Simple mode	26	0.107 (0.019, 0.194)	0.233					
		Weighted mode	26	0.102 (0.019, 0.187)	0.233					
Salicylic acid	Acetoacetate	Inverse variance weighted	26	−0.136 (−0.184, −0.087)	0.005	−0.015 (0.069)	27.534 (0.489)	0%	25,569 (0.487)	1.56E−08
		MR Egger	26	0.008 (−0.124, 0.140)	0.953					
		Weighted median	26	−0.122 (−0.189, −0.055)	0.068					
		Simple mode	26	−0.124 (−0.238, −0.009)	0.291					
		Weighted mode	26	−0.113 (−0.215, −0.012)	0.276					
	Creatinine	Inverse variance weighted	26	−0.094 (−0.140, −0.048)	0.041	−0.002 (0.774)	27.543 (0.470)	0%	25.683 (0.425)	4.67E−10
		MR Egger	26	−0.061 (−0.183, 0.061)	0.621					
		Weighted median	26	−0.140 (−0.203, −0.077)	0.027					
		Simple mode	26	−0.165 (−0.283, −0.047)	0.174					
		Weighted mode	26	−0.162 (−0.265, −0.059)	0.128					
	Omega−3 fatty acids	Inverse variance weighted	23	0.123 (0.061, 0.185)	0.046	0.006 (0.582)	27.569 (0.395)	4%	25.477 (0.380)	1.04E−03
		MR Egger	23	0.022 (−0.141, 0.186)	0.892					
		Weighted median	23	0.145 (0.056, 0.234)	0.105					
		Simple mode	23	0.229 (0.057, 0.401)	0.196					
		Weighted mode	23	0.303 (0.153, 0.453)	0.056					
	Triglycerides in	Inverse variance	25	0.114 (0.066, 0.162)	0.018	0.003	34.933 (0.152)	0%	31.941	7.54E−08

	very large HDL	weighted				(0.751)			(0.160)	
		MR Egger	25	−0.031 (−0.161, 0.099)	0.813					
		Weighted median	25	0.101 (0.029, 0.174)	0.164					
		Simple mode	25	0.054 (−0.091, 0.199)	0.715					
		Weighted mode	25	0.054 (−0.085, 0.192)	0.703					

MR, mendelian randomization; NSAID and Salicylic acid, common pain medications; IVW, inverse-variance weighted; NSNPs, number of single nucleotide polymorphisms; Beta, mendelian randomization effect estimate; CI, confidence interval; RSSobs, residual sums of squares of observations;

Figure S1. Scatterplot of Mendelian randomization (MR) estimates between NSAID, Salicylic acid and gut microbiota.

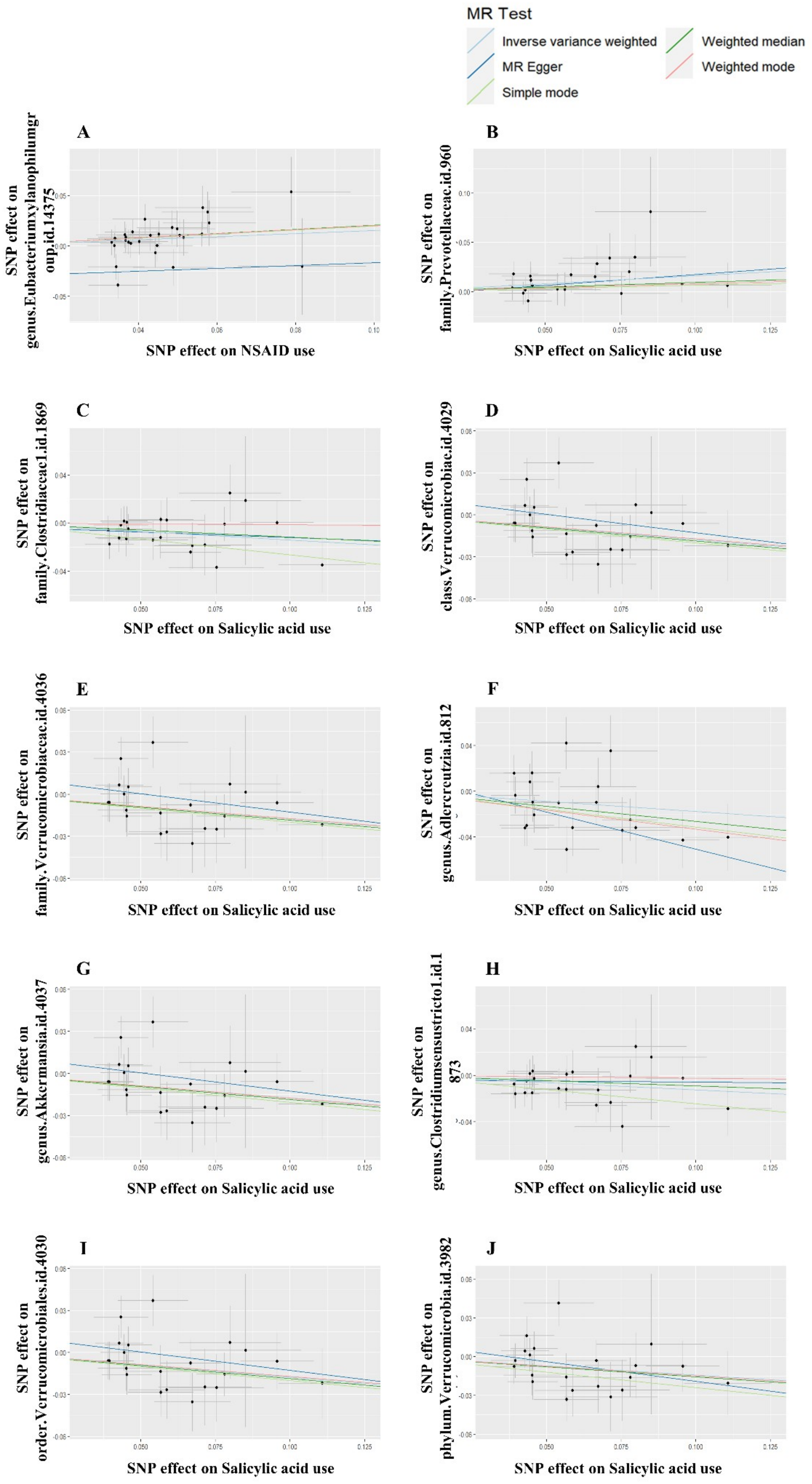

Figure S2. Scatterplot of Mendelian randomization (MR) estimates between the four pain medications studied and circulating metabolites.

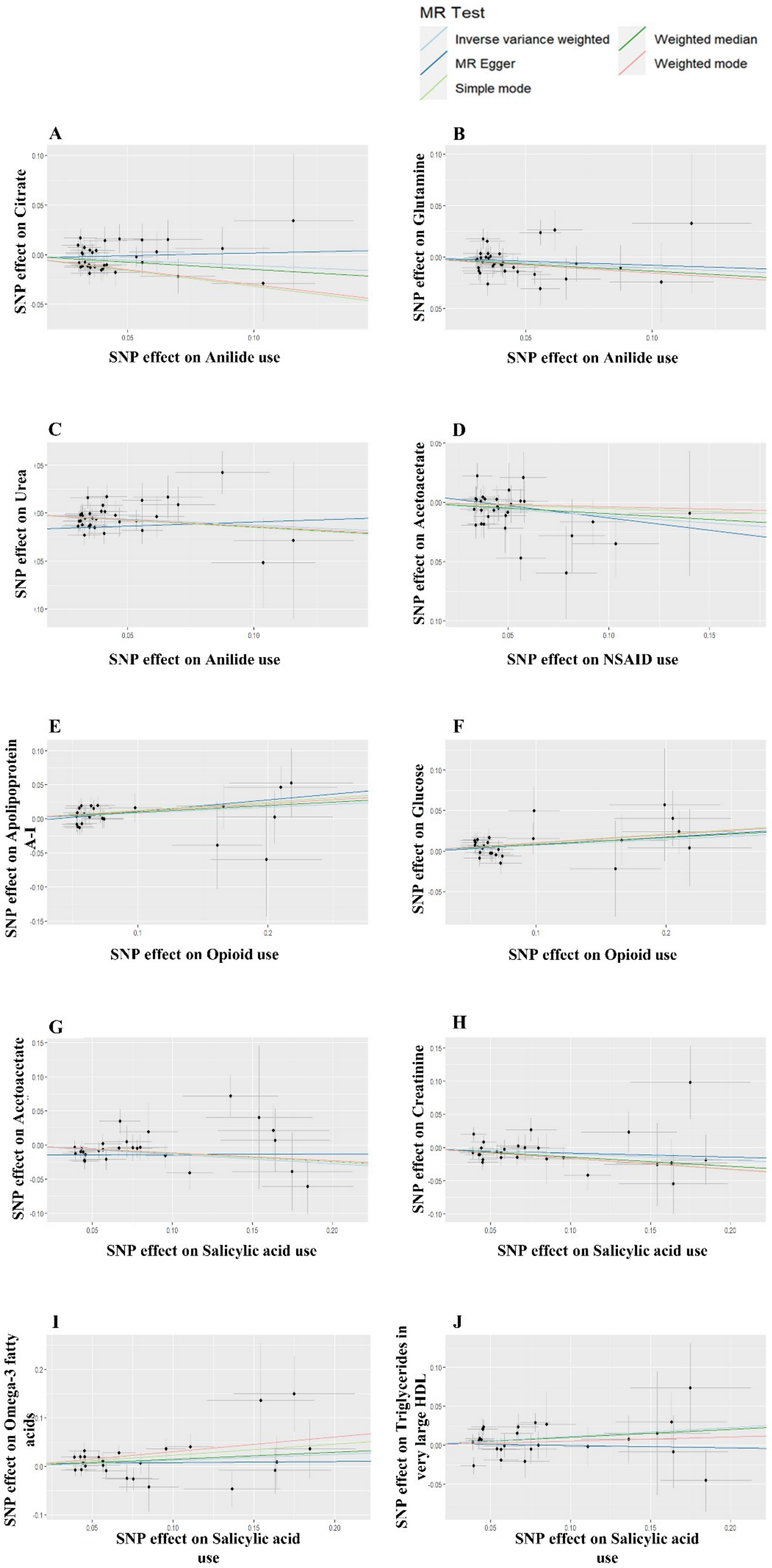

Figure S3. Leave-one-out analysis of inverse-variance weighted (IVW) estimates between NSAID, Salicylic acid use, and genetically determined gut microbiota traits ($p < 1 \times 10^{-5}$).

Black dots indicated the IVW estimates (raw beta) after leaving a single SNP in turns. Red dots indicated the pooled IVW estimate (raw beta). Horizontal lines indicated the range of 95% confidence interval.

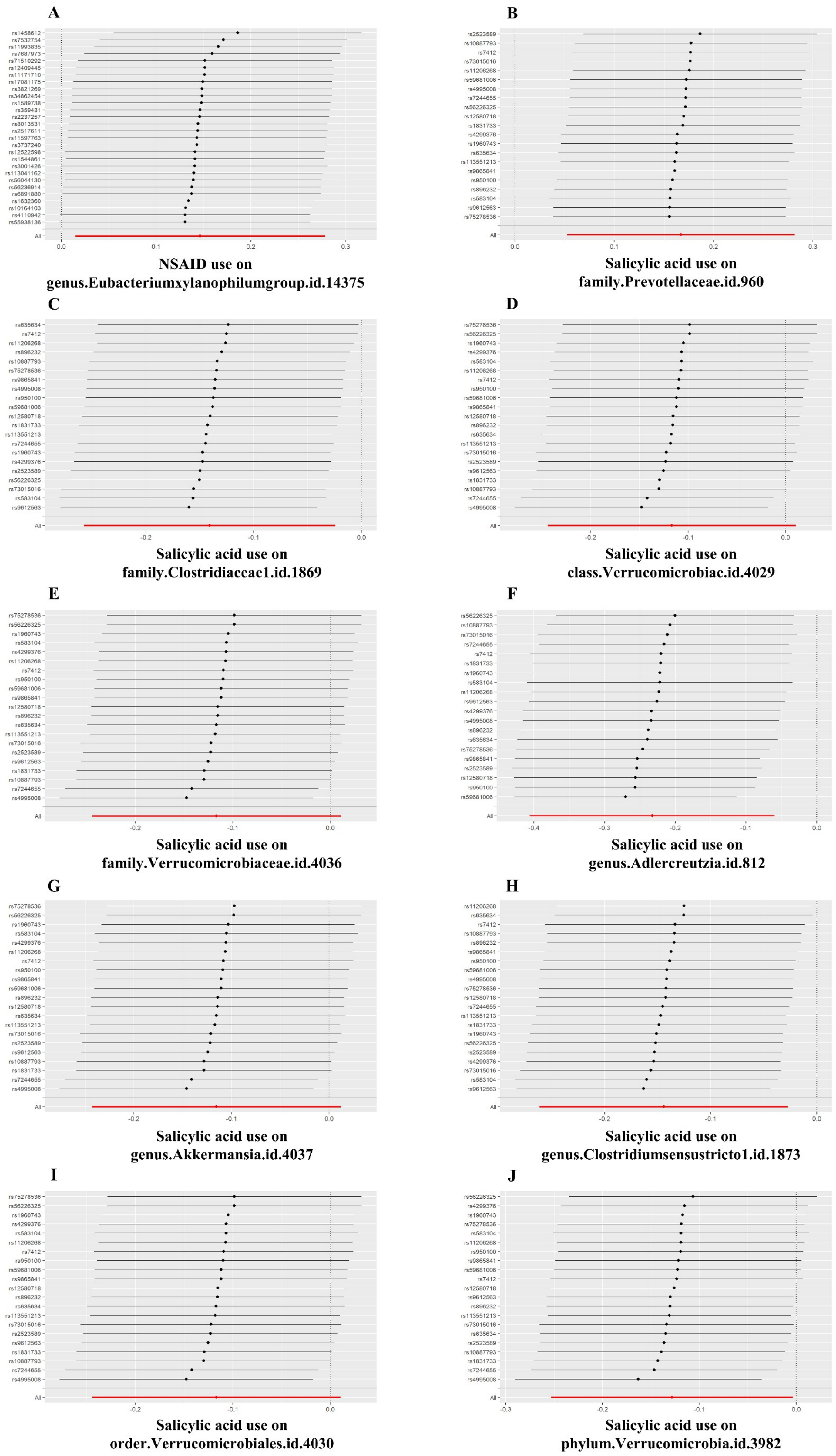

Figure S4. **Leave-one-out analysis of inverse-variance weighted (IVW) estimates between four prescription pain medications and genetically determined circulating metabolites traits ($p < 1 \times 10^{-5}$).**
Black dots indicated the IVW estimates (raw beta) after leaving a single SNP in turns. Red dots indicated the pooled IVW estimate (raw beta). Horizontal lines indicated the range of 95% confidence interval.

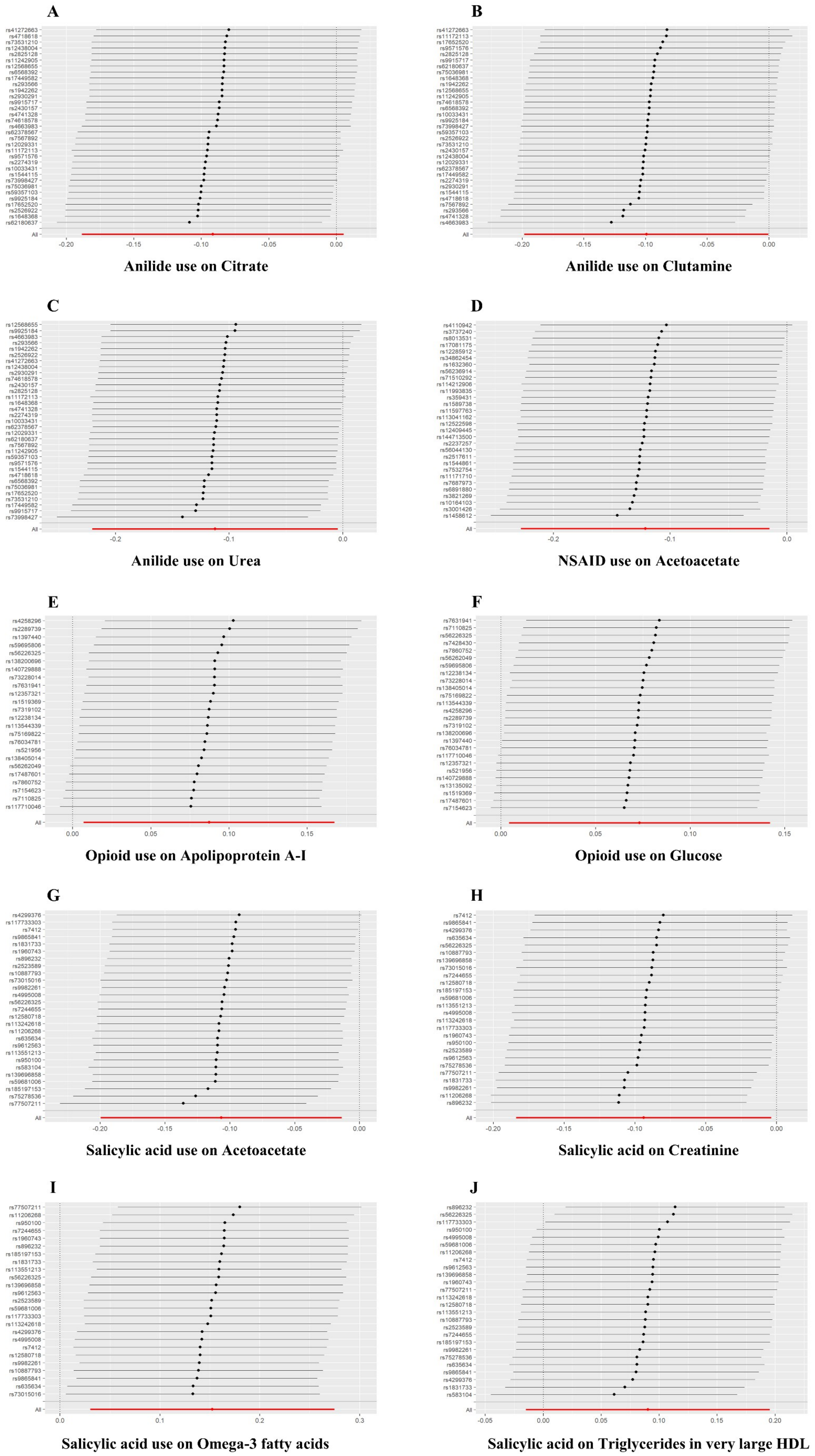

Supplement: Uncited Supplementary Material 1. [file jmm-74-02028-s001.pdf]
